# Engaging Australian Aboriginal narratives to challenge attitudes and create empathy in health care: a methodological perspective

**DOI:** 10.1186/s12909-016-0677-2

**Published:** 2016-06-02

**Authors:** Toni Wain, Moira Sim, Dawn Bessarab, Donna Mak, Colleen Hayward, Cobie Rudd

**Affiliations:** Edith Cowan University, 270 Joondalup Drive, Joondalup, WA 6027 Australia; University of Western Australia, Centre for Aboriginal Medical and Dental Health, 35 Stirling Hwy, Crawley, WA 6009 Australia; The University of Notre Dame Australia, 32 Mouat St, Fremantle, WA 6959 Australia; Edith Cowan University, Kurongkurl Katitjin, 2 Bradford St, Mount Lawley, WA 6050 Australia

**Keywords:** Study protocols, Indigenous methodology, Health professionals’ education, Narratives, Attitudes, Empathy

## Abstract

**Background:**

Unconscious bias and negative attitudes towards minority groups have detrimental effects on the way health care is, or is not, provided to these groups. Recognition of racist attitudes and behaviours as well as understanding clients’ experiences of health and health care are pivotal to developing better health care strategies to positively impact on the quality and safety of care provided to Indigenous people.

Indigenous research demands inclusive research processes and the use of culturally appropriate methodologies. This paper presents a methodological account of collecting narratives which accurately and respectfully reflect Aboriginal Australians’ experiences with health care in Western Australia. The purpose of these narratives is to provide health students and professionals with an opportunity to ‘walk-in the shoes’ of Aboriginal people where face-to-face interaction is not feasible.

**Methods:**

With the incorporation of Indigenous peoples’ voices being an important link in cultural safety, the project was led by an Indigenous Reference group, who encouraged active participation of Aboriginal people in all areas of the project. Using a phenomenological approach and guided by the Indigenous Reference group, yarning data collection was implemented to collect stories focusing on Aboriginal people’s experiences with health care services. An open-access, on-line website was established to host education resources developed from these “yarns”.

**Results:**

Yarning provided a rich source of information on personal experiences and encouraged the story provider to recognise their facilitative role in the research process. While the methodology used in this project was lengthy and labour-intensive it afforded a respectful manner for story collection and highlighted several innate flaws when Western methods are applied to an Indigenous context.

**Conclusion:**

Engagement of an Indigenous Reference Group was pivotal to designing an appropriate methodology that incorporated the voices of Aboriginal people in a multimedia resource of Aboriginal narratives. However further research is warranted to understand how the resources are being used and integrated into curricula, and their impact on students and health care outcomes.

## Background

Australian Indigenous people constitute 3 % [1] of the Australian population. Despite a national priority of “Closing the Gap” between the health outcomes of Indigenous and non-Indigenous people [2] the health of Indigenous Australians^1^ is among the worst in the developed world [3].

The reasons for this are complex, however social disadvantage and marginalisation from mainstream society are still largely implicated [4], including a lack of culturally appropriate health services [5] and racism [6–8]. The cultural differences between Indigenous Australians and health care providers have been recognised since the 1970s [9] but still Indigenous Australians report being subjected to racism resulting in poor health and wellbeing [10]. Health providers often hold unconscious biases and stereotypes [11] which can have devastating consequences to health care recipients [7, 12].

Cultural competency training for health providers has been suggested as a solution to improve racial disparity in health care. However, cultural competency training has also been criticised as a mechanism to improve the health of minority groups. Carpenter and colleagues [13] suggest that this approach does not acknowledge diversity within groups and assumes culture is static. Cultural competency encourages stereotyping, as knowledge is based on broad population level data [14, 15] and “competency” assumes an endpoint is achievable [14]. In recognising a population as disadvantaged, “we inadvertently and unavoidably label that population as inherently disadvantaged” [16] resulting in disadvantage being seen as the group’s characteristic rather than the result of historical events and external factors which may have had an impact on that group.

As a result of emerging patient-centred care and narrative-based medicine, a reflective and transformational approach to address racism has been proposed. The concept of cultural safety as described by Ramsden and Spoonley [17] emphasises awareness of the dominant hegemony and reflection on how this impacts the health care and health outcomes of minority groups. Recognition of unconscious bias and racist attitudes and behaviours should therefore be pivotal in developing better health care strategies [18]. Transformational learning occurs as a result of a “disorientating dilemma”, a situation that calls into question personal beliefs and challenges an individual’s “frame of reference” [19]. As a result of this cognitive disequilibrium and critical reflection, a more open, inclusive attitude towards patient-centred care can emerge [20]. However as Kumashiro [21] highlights the root of oppression does not reside solely in how individuals think, feel and behave towards others. Other sociocultural barriers to care such as organisational and structural processes and policies of the dominant culture also need to be addressed to enhance access, quality and cultural safety of health care for minority groups [22].

In teaching and learning a number of successful strategies have been used to reduce unconscious bias and stereotyping, some of which include: teaching people to recognise their own unconscious biases [11], stimulating disagreement in a non-threatening environment and emphasising the difference between behaviours and beliefs [23]. To facilitate self-reflection, educators use narratives and case studies as triggers to prompt discussion and questions. The stories enable learners to experience a new reality and encourage reflection on their own assumptions and values as well as on issues of social justice [24].

Other successful tactics have included demonstrating unconscious stereotyping to facilitate recognition of its existence and emphasise greater perspective-taking and empathy [11, 23] as well as integrating the patient perspective [25, 26]. Empathy is an appreciation or imagination of another person’s emotions [27] which involves a cognitive element (taking the perspective of another) and an emotional element [28]. Taking the perspective of another will lead to empathy for that person [29] and will increase the importance placed on an individual’s welfare, which in turn can motivate people to modify their behaviours and attitudes. It has been proposed that empathy can bridge cultural differences by providing a means to integrate an attitude of openness to diversity with the appropriate knowledge and skills to work successfully with people from other cultures [29].

In psychology and health promotion domains, the use of narrative pedagogy is a growing area of research. Narratives are particularly useful when strong attitudes, which are particularly resistant to change, are to be challenged. Identifying with characters in the narrative reduces the amount and effectiveness of counter-arguing [30]. Narratives also allow for activities, events and the significance of experiences to be more readily recounted [31] and can help illustrate cultural values [32].

A narrative is unlikely to be comprehended from a single point of view or a single moment [31] as interpretation is likely to be shaped by personal experiences and opinions. Furthermore, the reading response theory, which expands on the inter-subjectivity properties of narratives, pays attention to the phenomenological act of following a story, which allows the reader to take into account the underlying structure of the story as well as making sense of it [32].

In this paper we present a descriptive study of a method for collecting narratives from Aboriginal Australians in Western Australia regarding their experiences with health care and using these to create open-access, on-line learning resources. These resources can be used by educators as triggers for classroom discussions on unconscious bias both amongst the students themselves and in the wider community, encouraging self-reflection on assumptions and values as well as issues of social justice [24].

## Methods

### Key considerations in Aboriginal Indigenous research

Nguyen [26] advocates that incorporating the voices of Indigenous people is an important link in promoting cultural safety, and as such, inclusive approaches were central to this project. The six values of reciprocity, respect, equality, responsibility, survival and protection and spirit and integrity that lie at the heart of the Australian National Health and Medical Research Council’s guidelines for ethical conduct in Aboriginal and Torres Strait Islander Health Research [33] underpinned all aspects of this project. Aboriginal people were active participants, with roles as leaders, researchers, advisors and members of the Indigenous Reference Group (IRG), as well as providers of the narratives.

At the start of the project, an Indigenous Reference Group including three male and six female leaders in Aboriginal health and research was established to determine the methodology for story collection and recruitment of story providers. The IRG also identified the themes from the narratives and provided discussion points from the narratives and scenarios for educators to incorporate into lesson plans.

Ethics approval was obtained from both the Edith Cowan University Human Research Ethics Committee and Western Australian Aboriginal Health Ethics Committee (WAAHEC). WAAHEC approval required letters of support from the Geraldton Regional Aboriginal Medical Service, North and South Metropolitan Area Health Services, to recruit Aboriginal story providers in their area.

#### Indigenous methodology

Indigenous methodology has been described as research that uses “techniques and methods drawn from the traditions of those people” [34]. Yarning is an Indigenous cultural form of conversation and has been shown (by Bessarab, a member of the IRG) to be a rigorous and credible research method when gathering data from Indigenous people [35]. This method was suggested by the IRG for this project. Yarning involves “an informal and relaxed discussion though which both the researcher and the participant journey together visiting places and topics of interest relevant to the research” ([35] p38).

Integral to the yarning process is the establishment of a relationship between the story collector and the story provider through a “social yarn” (informal) before moving into the “research yarn” (purposeful conversation with a defined beginning and end). The yarn relies on the story provider to determine what parts of their story to reveal and what to leave out, while the story collector aims to draw out the parts of the story they are interested in [35].

#### Phenomenological approach

Morris [36] suggests that thinking with stories allows the narrative to work on the reader or viewer. He sets this against the institutionalised Western practice of thinking about stories where narratives are considered an object. Hence, the use of formal phenomenological steps to analyse and describe the themes within the narratives seemed to be counterintuitive. The narratives are presented as an agent for students’ meaning-making and self-reflection on the experiences of the Aboriginal story providers. Furthermore the objectifying and compartmentalising of experience flies in the face of the holistic nature of Indigenous epistemologies [37].

To prompt students to consider the values, beliefs and cultural perspective of both the story provider and themselves we asked the Indigenous Reference Group to develop optional questions and discussion points for educators.

#### Preparation for story collection

We recruited and trained seven Aboriginal and non-Aboriginal male and female story collectors in the use of “yarning” as a data collection tool. The training consisted of a 2-day workshop conducted by three members of the IRG.

The IRG identified that the term “story teller” has a particular significance in Aboriginal culture; hence the term “story provider” was used. Story providers were recruited by the Indigenous Reference Group members and story collectors through their personal contacts or by snowball sampling. A total of twenty-one Aboriginal story providers were recruited, the majority of whom lived in rural and remote areas of the Murchison in Western Australia (WA). However, the stories relate to experiences across WA from metropolitan areas of Perth to regional towns and rural and remote settings. All interviews from story providers were developed into narrative resources as this was considered to be respectful of their participation in the project.

#### Story collection process

Prior to recording their conversation (yarn), the story collectors explained the purpose and process for story collection with the story providers. To ensure that the story collection process was culturally appropriate and to provide a feeling of safety, the story provider chose the venue for this process and had the choice of whether their story was recorded on video or digitally recorded for transcription to text. Furthermore, since this process could potentially be traumatic, a list of counselling services with knowledge of the project was made available.

A two-step written consent process was used: the first to obtain consent at the interview for recording the interview, on video or digitally recorded for transcription to text; and the second to obtain informed consent for the use of the final stories derived from the story providers’ interviews. This last step allowed story providers to view the product before being asked to sign the consent form.

#### Story editing

The yarning methodology provided rich descriptions of the story providers’ experiences of health care. However, many of the audio and video tapes were more than an hour long. To maximise utility for teaching purposes, videos and transcripts from the audio recordings were edited to reflect a single incident or theme. Deconstructing the yarn into discrete incidents that can practically be used in teaching and learning potentially loses the flow and nuance of the story provider’s yarn. While this was unavoidable it was mitigated through the involvement of the IRG in unpacking the individual stories and ensuring that the story providers approved of the way their yarn was edited.

#### Website resources

An open-access website, requiring registration was developed for the project [38]. The website contains a total of 41 separate narratives embedded as YouTube videos and/or transcripts, depending on the story provider’s preference.

Four overarching themes from all of the narratives were identified by the IRG members and developed into scenarios written by Aboriginal playwright David Milroy and professionally produced. These key themes are communication, stereotypes around perceived drunkenness, passing on and a story reflecting the experiences of a member of the Stolen Generation. The term ‘Stolen Generation’ refers to approximately 100,000 Aboriginal children who were forcibly removed or taken under duress from their families by police or welfare officers from the late 1800s into the 1960s [39].

The Project Team and IRG developed facilitation guides which support the narrative and scenario resources and aid in classroom discussions. The object of the facilitation guides was to explain how the narratives were collected and scenarios developed and to provide questions and discussion points that could be used in teaching and learning. To assist educators in locating topic-based narratives, we included a search function which includes story provider sex, age group the story relates to, clinical and health system topic and type of media.

## Results

### Cultural issues

There were a number of controversial topics which arose where Western research methods were incompatible with a culturally respectful approach. The first, and the most obvious one, was having adequate time for the project; engaging with Indigenous people, building a relationship and trust called for a timeframe not necessarily congruent with the duration of the project’s funded period. Fortunately the project funder, the Australian Government Office for Learning and Teaching, agreed to extend the duration of the project from 2 years to 2-and-a-half years.

Second, in a number of Aboriginal language groups, there are cultural reasons for avoiding showing the image of Aboriginal people who have died [40]. Deciding how the project would manage a participant’s story should they pass on during or after the project when individual consent had been obtained was central to the discussions with the IRG. A critical part of obtaining consent from story providers was ensuring that they understood that if removal of video was requested at any point we would remove it from the website but we could not guarantee all copies of the videos had been destroyed as educators may download videos from the website. In an attempt to reduce this risk the videos are available on a USB stick but only sent to a named person who has agreed to destroy the USB if a request was made by the story provider or a relative to remove a video narrative. Requesting website users to create a login with an e-mail address, ensures that in the event that a video needs to be removed, we are in a position to contact those who have potentially downloaded the video.

Third, the IRG suggested a reasonable cash payment for the story providers as their story could be considered a cultural product, however the ethics committees considered this to be an incentive. The issue of cultural intellectual property, its value and worth was never resolved and remains a concern in this kind of research. The IRG recognised that further discussion on this issue would hold up the project so a decision was made to offer story providers a $25 reimbursement for transport and other costs associated with attendance. Each story provider also received a transcript and DVD copy (where relevant) of their story to share with their family.

### Reflections on the methodology

Yarning provides a rich source of information on personal experiences and allows the story provider to set the pace and agenda encouraging them to recognise their facilitative role in the research process [41]. However as described by Bessarab and Ng’andu the inherent challenge for researchers in using yarning for data collection is trusting in the story telling process and knowing when to interrupt or gently steer the conversation to another area [35]. When the recordings of interviews were playing back two of the IRG members identified times when the story collector interrupted the yarn as the story provider paused to reflect on an important point. This was a particular issue for one of the non-Aboriginal story collectors and perhaps reflects cultural differences in tolerance for conversational silences which Mushin and Gardner define as “breaks in the flow of talk within and between turns of talk” [42].

### Advantages of the methodology

The epistemological differences between Western and Indigenous philosophies suggest there are innate flaws when Western methods are applied to an Indigenous context which could potentially produce results that are distorted and incorrect [43]. Engagement of the IRG provided an interface between these epistemological differences and created a foundation from which the Aboriginal narrative resources could be generated, guaranteeing that they were true to, and respectful of Aboriginal cultures. For example, the role of IRG as methodology designers and story provider recruiters provided solutions to the challenge of lack of trust between researchers and participants and the use of appropriate research discourse (e.g. story providers rather that story tellers), common concerns in indigenous participatory research [44].

The methodology role-modelled a genuine alliance with Indigenous people rather than a more common tokenistic approach to Indigenous participation in research such as superficial representation on steering committees [45]. The non-Indigenous researchers found working across cultures particularly enriching, as the trusting relationship with the IRG allowed for naïve questions about cultural issues and constructive feedback.

“Witnessing” the social determinants that affect people’s lives “by observing and acquiring firsthand knowledge” is important [46]. Medical students exposed to community engaged rural placements have been shown to have unique learning opportunities that are not necessarily available to their urban counterparts. Such placements provide them with a unique preparedness for clinical practice by increasing, amongst other things, cultural understanding [47]. Unfortunately it is not possible for all students to spend time in these communities and there is a risk of culturally unsafe students being intrusive. A narrative approach offers the “next best thing” by providing its audience with an opportunity to “walk-in-the shoes” of Indigenous people, to experience their feelings and values and imagine the world from their unique individual perspective. Of course the resources cannot replace the experience of interacting face-to-face with an Indigenous person or living and working in an Indigenous community or organisation. However, they can be used to enhance and supplement other, more theoretical learning resources and to better prepare student health professionals for clinical placements in which they are likely to encounter Indigenous clients and colleagues.

The methodology used in this project was lengthy and labour-intensive but it afforded a respectful manner for story collection and the consent process. These are not stories of a “inherently disadvantaged population” [16] but rather personal reflections that validate Indigenous Australians’ support for change and provide insights into a strong and resilient culture.

## Conclusion

This study involved the use of culturally appropriate process to collect and disseminate narrative resources in partnership with Aboriginal people. The development of future teaching and learning resources to challenge unconscious bias and stereotype would benefit from the use of Indigenous methodologies rather than Western centric approaches that reflect the dominant hegemony. This project encourages empathy and openness to cultural differences through critical reflection on health professionals’ own cultural identities rather than a static knowledge of a culture.

This study confirms that establishing Indigenous and non-Indigenous shared leadership for project governance and management is essential for building a rich multimedia resource of their experiences with health services. These resources have resonated with Australian educators and students signifying their potential to build capacity by challenging attitudes and cultivating deep and lasting empathy. Research is now needed that explores the level of empathetic change in health care students and practitioners following exposure to these resources.

## Abbreviations

IRG, Indigenous Reference Group; WA, Western Australia; WAAHEC, Western Australian Aboriginal Health Ethics Committee.

## Footnotes

1 We have referred to Australian “Indigenous” people (who include Aboriginal and Torres Strait Islander people) and Australian “Aboriginal” people. Since this was a federally funded project that aimed to influence health care education across Australia, we use the term “Indigenous” when describing existing national research and issues that relate to the broad Aboriginal and Torres Strait Islander population. However the Aboriginal people in Western Australia were the Indigenous Australians who contributed their stories to our project, hence we use the term “Aboriginal” in relation to the story providers.
